# Development of a New Radiation Shield for the Face and Neck of IVR Physicians

**DOI:** 10.3390/bioengineering9080354

**Published:** 2022-07-29

**Authors:** Toshimitsu Sato, Yoichi Eguchi, Chika Yamazaki, Takanobu Hino, Toshikazu Saida, Koichi Chida

**Affiliations:** 1 Department of Radiology, Yamagata University Hospital, 2-2-2 Iida-nishi, Yamagata 990-9585, Japan; tssato@med.id.yamagata-u.ac.jp (T.S.); zaki-chika@med.id.yamagata-u.ac.jp (C.Y.); t-hino@med.id.yamagata-u.ac.jp (T.H.); 2 Course of Radiological Technology, Health Sciences, Graduate School of Medicine, Tohoku University, 2-1 Seiryo, Sendai 980-8575, Japan; yoichi.eguchi.b4@tohoku.ac.jp; 3 Department of Central Radiology, Nara Prefecture Seiwa Medical Center, 1-14-16 Mimuro, Nara 636-0802, Japan; saitosh_555@mac.com; 4 Department of Radiation Disaster Medicine, International Research Institute of Disaster Science, Tohoku University, 468-1 Aramaki Aza-Aoba, Sendai 980-0845, Japan

**Keywords:** radiation protection and safety, fluoroscopy, interventional radiology (IVR), fluoroscopically guided interventional procedures, percutaneous coronary intervention (PCI), protective apron, face shield, radiation dose, X-ray examination, disaster medicine

## Abstract

Interventional radiology (IVR) procedures are associated with increased radiation exposure and injury risk. Furthermore, radiation eye injury (i.e., cataract) in IVR staff have also been reported. It is crucial to protect the eyes of IVR physicians from X-ray radiation exposure. Many IVR physicians use protective Pb eyeglasses to reduce occupational eye exposure. However, the shielding effects of Pb eyeglasses are inadequate. We developed a novel shield for the face (including eyes) of IVR physicians. The novel shield consists of a neck and face guard (0.25 mm Pb-equivalent rubber sheet, nonlead protective sheet). The face shield is positioned on the left side of the IVR physician. We assessed the shielding effects of the novel shield using a phantom in the IVR X-ray system; a radiophotoluminescence dosimeter was used to measure the radiation exposure. In this phantom study, the effectiveness of the novel device for protecting against radiation was greater than 80% in almost all measurement situations, including in terms of eye lens exposure. A large amount of scattered radiation reaches the left side of IVR physicians. The novel radiation shield effectively protects the left side of the physician from this scattered radiation. Thus, the device can be used to protect the face and eyes of IVR physicians from occupational radiation exposure. The novel device will be useful for protecting the face (including eyes) of IVR physicians from radiation, and thus could reduce the rate of radiation injury. Based on the positive results of this phantom study, we plan to perform a clinical experiment to further test the utility of this novel radiation shield for IVR physicians.

## 1. Introduction

Interventional radiology (IVR) procedures are increasingly being performed because of the significant advantages for patients [1,2,3,4,5,6]. However, IVR procedures are associated with increased radiation exposure and injury risk in both patients and IVR staff [7,8,9,10]. Many studies have evaluated the radiation dose to patients and IVR staff, and methods to reduce exposure [11,12,13,14,15,16,17,18]. We also previously evaluated exposure of patients and staff to radiation in our IVR laboratory [19,20,21,22,23,24,25,26].

In 2011, the International Commission on Radiological Protection significantly reduced the limit of occupational exposure of the eyes to radiation, from 150 to 100 mSv/5 years (i.e., 20 mSv/year) [27]. Furthermore, radiation eye injury (i.e., cataract) in IVR staff has also been reported [28,29]. It is crucial to protect the eyes of IVR physicians from X-ray radiation exposure [30,31,32,33,34,35]. Therefore, evaluation of the exposure of the eyes of IVR physicians to occupational radiation, and related protection, is important [36,37,38,39,40].

Lead (Pb) eyeglasses are useful for shielding the eyes against radiation [41,42,43]. Many IVR physicians use protective Pb eyeglasses to reduce occupational eye exposure. Despite the diversity in the thickness and shape of Pb eyeglasses, none offer complete protection against radiation exposure to the eyes of IVR physicians [44,45,46]. Therefore, we developed a unique face radiation shield that also protects the eyes. The device was designed to protect the neck and the left side of the face, including the left eye, of IVR physicians.

The purpose of this phantom study was to evaluate the radiation-protective effects of the novel shield in an IVR X-ray system.

## 2. Materials and Methods

### 2.1. Development of the Novel Radiation Shield

**Figure 1:**
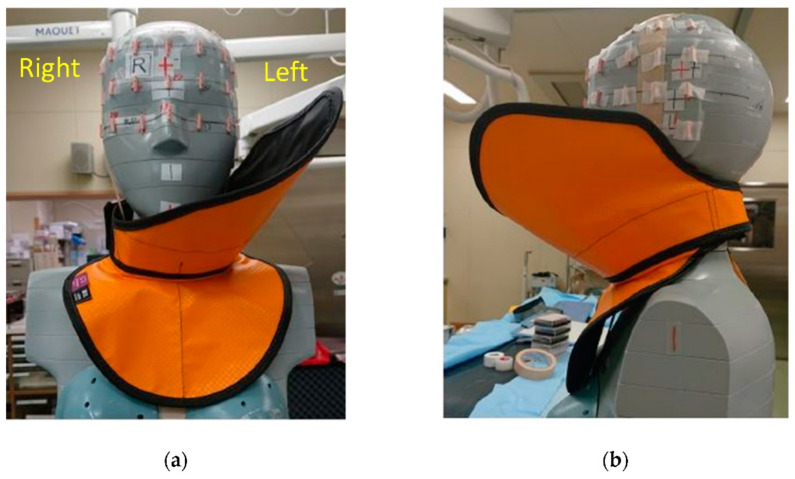
Photograph of the novel shield: (**a**) frontal view; (**b**) lateral view. The neck guard and face shield are fastened together to create a single device, which cannot be disassembled. The face shield is attached to the left side of the neck guard and protects the left side of the physicians’ neck and face from radiation. The novel shield was designed so that it does not obstruct IVR physicians’ field of vision.

**Figure 2:**
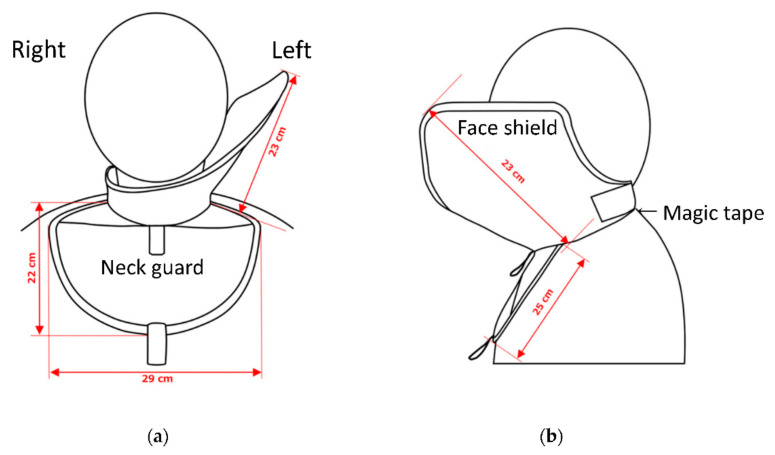
Schematic of the novel shield: (**a**) frontal view; (**b**) lateral view. The novel shield consists of a neck guard and face shield, which together comprise a single unit to promote stability and prevent misalignment. The shield is firmly attached behind the neck of the IVR physicians using Velcro to protect them from scattered radiation from the left side. The device consists of a neck guard and face shield designed using a 0.25 mm Pb-equivalent rubber sheet (nonlead protective sheet).

Figure 1 shows the novel radiation shield for IVR physicians. The device consists of a neck guard and face shield designed using a 0.25 mm Pb-equivalent rubber sheet (nonlead protective sheet, Figure 2). Pb-equivalent rubber sheeting is easy to handle and often used in personal protective aprons. The device is lightweight (0.65 kg). The neck guard and face shield are firmly connected and have adequate stability. The face shield was designed to mainly protect the left side of IVR physicians from scattered radiation.

### 2.2. Phantom Study

**Figure 3:**
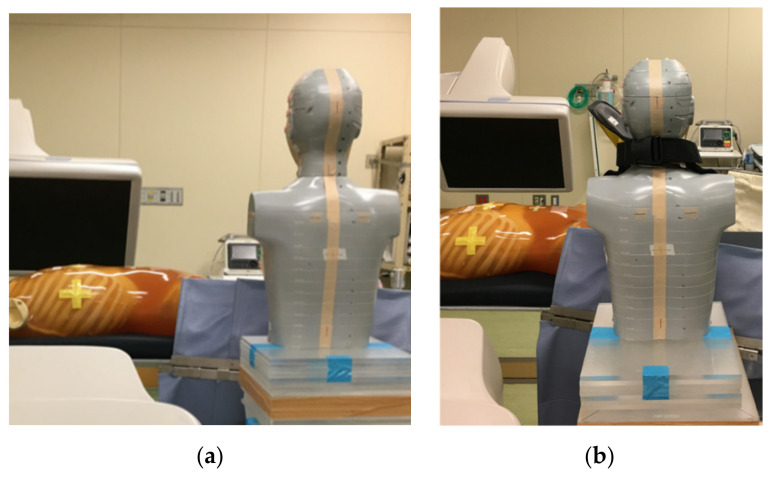
Experimental setup used for our phantom study (e.g., LAO60): (**a**) without novel shielding device; (**b**) with novel shielding device.

We conducted a phantom study at Yamagata University Hospital, Japan. Figure 3 displays the experimental setup used to simulate the typical settings for IVR procedures.

A digital cine angiography X-ray unit (an “under-tube” X-ray tube system) with a 16-in mode flat-panel detector (FPD) was used. Digital cine acquisitions were performed at 30 frames/s with a total duration of 150 s (30 s × 5). An automatic control system was used to set the X-ray exposure settings (i.e., kilovoltage and milliamperage) (Table 1).

We set the focus-to-image receptor (i.e., FPD) distance to 120 cm, and the height of the patient table to 92 cm. Five standard tube-viewing angles were used to simulate the typical settings for percutaneous coronary intervention (PCI) and cardiac catheterization: posteroanterior (PA), 60° left anterior oblique (LAO), 30° right anterior oblique (RAO), 30° RAO + 30° caudocranial (cranial), and 60° LAO + 30° craniocaudal (caudal).

A trunk phantom (PBU-60) was used to simulate the patient (Figure 3). A head phantom (THRA1) was used to simulate the IVR physician (Figure 3); it was placed 70 cm horizontally and 40 cm vertically from the central radiation beam on the patient table. This position is similar to that used by physicians during PCI at our hospital. The height of the head phantom was 165 cm; therefore, the eye of the phantom was approximately 150 cm above the floor. We did not use a ceiling-protecting Pb plate.

### 2.3. Dosimetry

**Figure 4:**
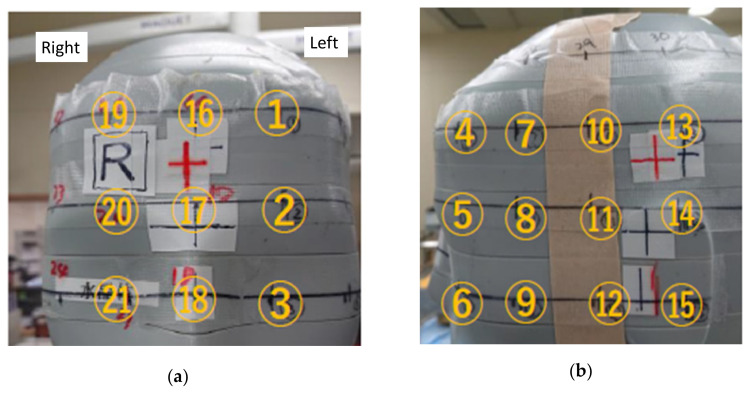

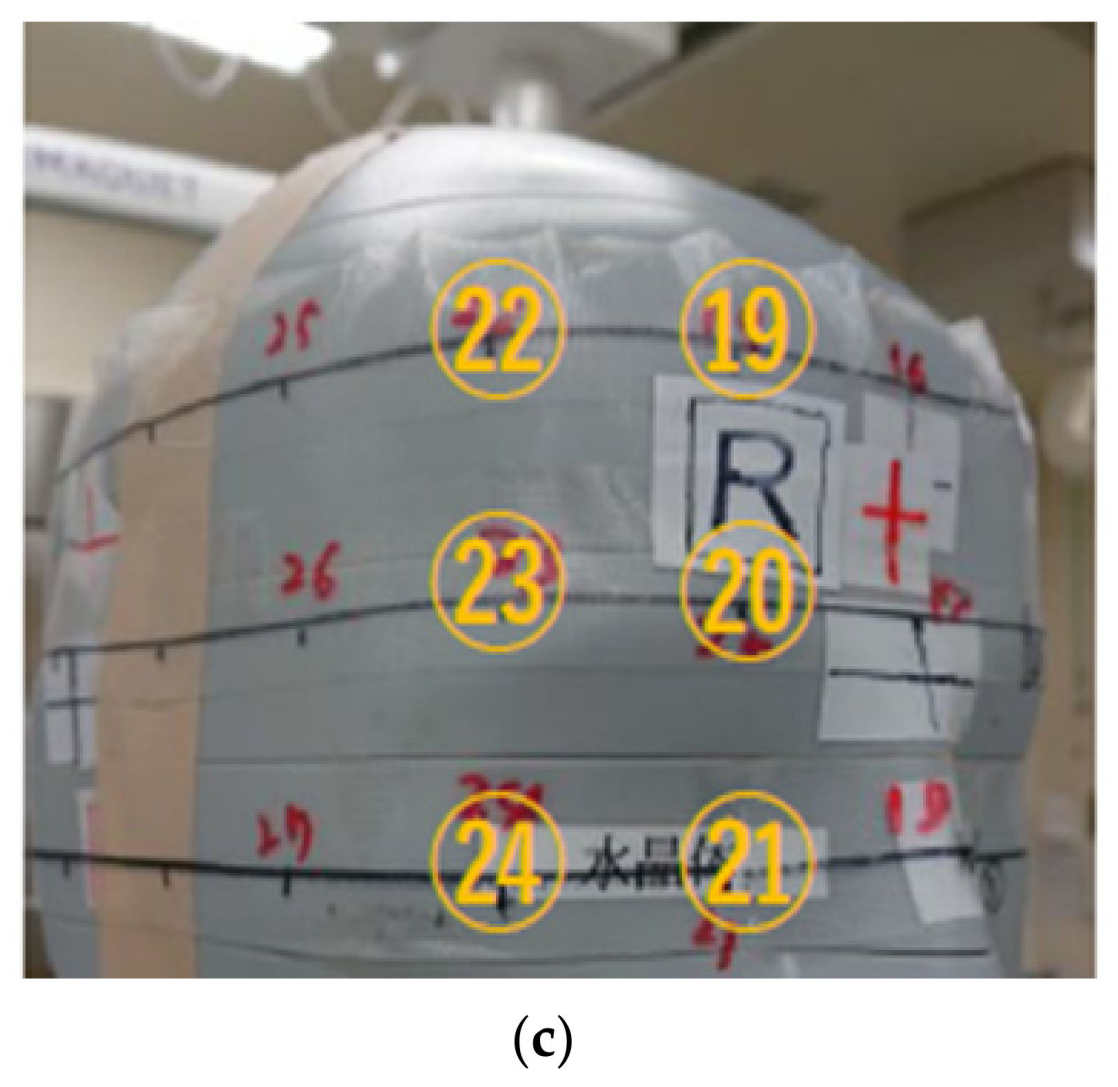
The 24 measurement points on the head of the phantom simulating the physician: (**a**) frontal view; (**b**) left lateral view; (**c**) right lateral view. Twenty-four dosimeters were attached to the points marked on the phantom’s surface (left eye: No. ③, right eye: No. **㉑**). The distance between the measurement points was 3 cm.

Scattered radiation from the trunk phantom representing the patient was measured using radiophotoluminescence dosimeters (RPLDs; GD-302M), with and without the novel radiation shield. Dose Ace FGD-1000 was used as the measurement/readout system. RPLDs were placed on the surface of the head phantom representing the physician at 24 locations, including the left (No. ③) and right (No. ㉑) eyes (Figure 4).

The background radiation dose was subtracted from the measurements, and the doses were calibrated. The average of three measurements was recorded for each X-ray viewing angle. Based on the doses measured with (D_with_) and without (D_without_) the novel radiation shield, we calculated the effectiveness of the radiation protection of the shield as: (D_without_ − D_with_)/D_without_ × 100%.

## 3. Results

Table 2 summarizes the results of our phantom study of the novel radiation shield. The scattered radiation doses were highest and lowest for the LAO views (LAO 60° and LAO 60° + CAU 30°) and RAO views (RAO 30° and RAO 30° + CRA 30°), respectively, for all measurements acquired without the novel radiation shield.

The scattered radiation doses were higher for the left side (No. ①–⑮) compared to the right side (No. ⑲–㉔) of the face.

**Figure 5:**
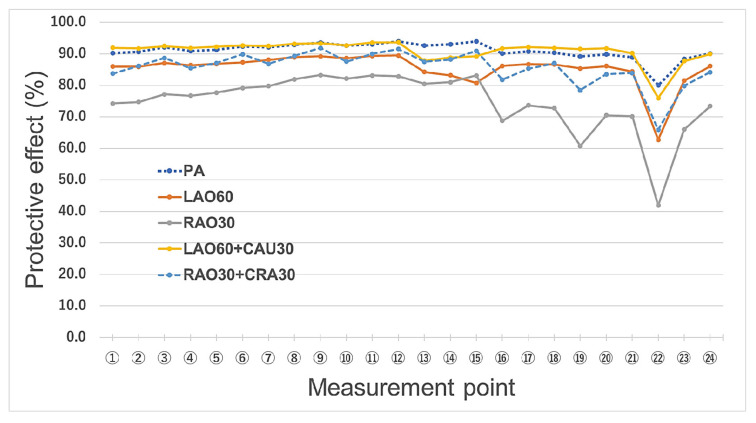
Protective effect of the novel radiation shield in the phantom study. Measurement point: Twenty-four dosimeters were attached to the points marked on the phantom’s surface (left eye: No. ③, right eye: No. ㉑) (See Figure 4). PA: posteroanterior, LAO60: 60° left anterior oblique, RAO30: 30° right anterior oblique, LAO60+CAU30: 60° left anterior oblique + 30° craniocaudal (caudal), RAO30+CRA30: 30° right anterior oblique + 30° caudocranial (cranial).

Figure 5 depicts the protective effect of the novel radiation shield. The radiation protection effectiveness of the novel radiation shield was greater than 80% at almost all measurement points, except RAO 30°, at which the effectiveness was slightly lower. The average radiation protection effectiveness of the novel device for the five viewing angles were 87.5% and 83.6% for the left (No. ③) and right (No. ㉑) eyes, respectively.

## 4. Discussion

It is crucial to evaluate exposure of patients and healthcare workers to radiation during radiological examinations, especially IVR [47,48,49,50,51,52]. Despite the importance of protecting IVR physicians from occupational radiation exposure, no ideal radiation shield exists [53,54,55,56]. Although many devices protecting against radiation are available, none offer complete protection, especially for IVR physicians [54,57,58].

We developed a novel radiation shield to protect the face of IVR physicians (Figure 1 and Figure 2). This device is lightweight and comfortable to wear and has a unique design that protects the face (including the eyes) of IVR physicians. To provide stability and prevent misalignment, the face shield is firmly connected to the neck guard as a single component (Figure 1 and Figure 2). The device also allows IVR physicians to have a full field of vision. The face shield is connected to the left side of the face because most occupational radiation exposure to IVR physicians occurs from that side.

At almost all measurement points, the radiation protection of the shield was greater than 80%, which confirms its usefulness for IVR physicians. However, slightly lower effectiveness (<80%) was observed for the RAO view and No. ㉒. Thus, the protective effects of the device were slightly reduced in the RAO view compared to the other views. However, compared to the left side of the face, the doses of radiation delivered to the right side are nonetheless small, such that the device would still be effective for protecting IVR physicians from occupational radiation exposure. Similarly, the protection at No. ㉑ (right eye) was relatively low (i.e., 70.5%) at RAO30; however, this is unlikely to be a problem because the radiation doses delivered to this area are also small.

Radiation exposure to physicians is greater in the LAO compared to the RAO view because of the higher levels of scattered radiation (from the patient to the physician) in the former view. Occupational radiation protection of the eyes is crucial for IVR physicians, and Pb eyeglasses are often used for this purpose. Lightweight and comfortable Pb eyeglasses (0.07 mm Pb-equivalent) are often preferred by IVR physicians because of the prolonged duration of IVR procedures. However, the radiation-shielding effect of 0.07 mm Pb-equivalent eyeglasses is inadequate (45–60%). Although the radiation-shielding effect of 0.75 mm Pb-equivalent eyeglasses (~80%) is superior to that of 0.07 mm Pb-equivalent eyeglasses, the latter glasses are heavy and uncomfortable, which makes them unsuitable for use by IVR physicians.

Our novel shield provides eye radiation protection of above 80% on average (left eye, ③: 87.5%, right eye, ㉑: 83.6%), which is superior to that of Pb eyeglasses.

Generally, the distance between the left side of the IVR physician and the scattered radiation source (i.e., the patient) is small, such that more scattered radiation is received by the left than the right side of the physician [42,43,59]. Therefore, our novel radiation shield was developed to protect the left side of the IVR physician’s head.

IVR physicians are also potentially at higher risk of radiation-induced brain tumors compared to the general population [60,61,62]. Roguin et al. reported a higher rate of tumors on the left compared to the right side of the brain in IVR physicians, which they attributed to the higher radiation dose to the left side of the head (because it is nearer to the primary X-ray beam and exposed to more scattered radiation) [63]. The novel shield was designed to protect particularly the left side of the head of IVR physicians, and thus may reduce the risk of radiation-induced brain tumors.

Currently, the novel shield is available only in a single size; small and large sizes may also be needed. The novel shield protects only the face and neck of IVR physicians. Therefore, other radiation shields (e.g., a protective apron) are also required.

Further studies comparing the eye-protective effect of our novel radiation shield with that of protective Pb glasses (using the same radioactive source in the same environment) may be needed. This study using phantoms introduces our novel shield for the face and neck of IVR physicians, but further investigation is required in clinical settings to fully test the shield.

## 5. Conclusions

We performed a phantom study to investigate the protective effects against radiation of a novel shield for the face and eyes of IVR physicians and found it to be highly effective (>80% protection) under almost all measurement conditions. The novel shield can reduce the radiation dose by more than 80% without the use of Pb eyeglasses and offers equivalent or superior protection compared to Pb eyeglasses.

A large amount of scattered radiation reaches the left side of IVR physicians. The novel radiation shield effectively protects the left side of the physician from this scattered radiation. Thus, the device can be used to protect the face and eyes of IVR physicians from occupational radiation exposure. The novel device will be useful for protecting the face (including eyes) of IVR physicians from radiation, and thus could reduce the rate of radiation injury. Based on the positive results of this phantom study, we plan to perform a clinical experiment to further test the utility of this novel radiation shield for IVR physicians.

## Figures and Tables

**Table 1:** X-ray exposure setup used in our study.

Tube-Viewing Angles	Tube Kilovoltage (kV)	Tube Milliamperage (mA)	Additional Copper Filter (mm)
60° left anterior oblique	74	320	0.3
30° right anterior oblique	83	320	0.3
Posteroanterior	74	320	0.3
60° left anterior oblique +30° craniocaudal	74	400	0.3
30° right anterior oblique +30° caudocranial	79	320	0.3

**Table 2:** Summary of the phantom study.

	Posteroanterior			60° Left Anterior Oblique			30° Right Anterior Oblique			60° Left Anterior Oblique +30° Craniocaudal			30° Right Anterior Oblique +30° Caudocranial		
^1^ MP	^2^ Without	^3^ With	^4^ *PE*	^2^ Without	^3^ With	^4^ *PE*	^2^ Without	^3^ With	^4^ *PE*	^2^ Without	^3^ With	^4^ *PE*	^2^ Without	^3^ With	^4^ *PE*
	(μGy)	(μGy)	*(%)*	(μGy)	(μGy)	*(%)*	(μGy)	(μGy)	*(%)*	(μGy)	(μGy)	*(%)*	(μGy)	(μGy)	*(%)*
①	6918	671	*90.3*	12,600	1764	*86.0*	3058	786	*74.3*	15,512	1250	*91.9*	4147	673	*83.8*
②	7673	716	*90.7*	13,451	1894	*85.9*	3207	809	*74.8*	16,460	1352	*91.8*	4289	591	*86.2*
③	8065	639	*92.1*	14,470	1861	*87.1*	3442	785	*77.2*	17,585	1319	*92.5*	4495	508	*88.7*
④	7821	706	*91.0*	13,510	1839	*86.4*	3304	770	*76.7*	15,799	1283	*91.9*	4491	653	*85.5*
⑤	8320	718	*91.4*	14,175	1867	*86.8*	3490	778	*77.7*	16,614	1284	*92.3*	4568	587	*87.1*
⑥	8473	640	*92.4*	15,145	1917	*87.3*	3645	760	*79.2*	17,821	1304	*92.7*	4786	484	*89.9*
⑦	8368	658	*92.1*	13,796	1640	*88.1*	3342	675	*79.8*	16,185	1215	*92.5*	4840	636	*86.9*
⑧	8686	620	*92.9*	14,510	1596	*89.0*	3637	657	*81.9*	17,103	1161	*93.2*	4865	516	*89.4*
⑨	9096	588	*93.5*	16,667	1789	*89.3*	4038	674	*83.3*	19,216	1281	*93.3*	5168	421	*91.9*
⑩	8074	592	*92.7*	13,757	1558	*88.7*	3167	566	*82.1*	15,833	1159	*92.7*	4621	574	*87.6*
⑪	8533	590	*93.1*	14,800	1569	*89.4*	3620	609	*83.2*	17,066	1088	*93.6*	4924	490	*90.1*
⑫	9534	573	*94.0*	16,966	1777	*89.5*	4080	699	*82.9*	19,278	1216	*93.7*	5319	449	*91.6*
⑬	7731	569	*92.6*	13,258	2080	*84.3*	2879	561	*80.5*	14,937	1819	*87.8*	4509	563	*87.5*
⑭	8406	581	*93.1*	14,764	2477	*83.2*	3274	620	*81.1*	15,837	1771	*88.8*	4664	547	*88.3*
⑮	8772	530	*94.0*	15,916	3059	*80.8*	3730	629	*83.1*	17,348	1869	*89.2*	4968	450	*90.9*
⑯	6353	627	*90.1*	11,267	1555	*86.2*	2714	847	*68.8*	13,699	1130	*91.7*	3735	680	*81.8*
⑰	6669	618	*90.7*	12,338	1634	*86.8*	2875	757	*73.7*	14,677	1153	*92.1*	3765	553	*85.3*
⑱	6283	607	*90.3*	12,998	1733	*86.7*	2822	769	*72.8*	15,090	1228	*91.9*	3891	504	*87.0*
⑲	5652	610	*89.2*	9515	1392	*85.4*	2470	968	*60.8*	12,094	1025	*91.5*	3380	726	*78.5*
⑳	5536	558	*89.9*	10,010	1385	*86.2*	2585	763	*70.5*	11,758	971	*91.7*	3387	556	*83.6*
㉑	3563	393	*89.0*	6026	940	*84.4*	1905	568	*70.2*	6548	640	*90.2*	2493	400	*84.0*
㉒	1498	298	*80.1*	1693	631	*62.7*	1050	610	*41.9*	1827	439	*76.0*	1346	458	*65.9*
㉓	3183	369	*88.4*	4071	754	*81.5*	1782	605	*66.0*	4146	510	*87.7*	2155	434	*79.8*
㉔	4118	405	*90.2*	6742	935	*86.1*	2298	611	*73.4*	6144	616	*90.0*	2778	438	*84.2*

^1^ MP: Measurement point. ^2^ Without: Doses measured without the novel radiation shield (The average of three measurements was recorded). ^3^ With: Doses measured with the novel radiation shield (The average of three measurements was recorded). ^4^ *PE*: Protective effect, (D_without_ − D_with_)/D_without_ × 100%.

## Data Availability

Not applicable.
